# Pitch Processing Can Indicate Cognitive Alterations in Chronic Liver Disease: An fNIRS Study

**DOI:** 10.3389/fnhum.2020.535775

**Published:** 2020-10-08

**Authors:** Geonsang Jo, Young-Min Kim, Dae Won Jun, Eunju Jeong

**Affiliations:** ^1^Daehong Communications Inc, Seoul, South Korea; ^2^Graduate School of Technology and Innovation Management, Hanyang University, Seoul, South Korea; ^3^College of Interdisciplinary Industrial Studies, Hanyang University, Seoul, South Korea; ^4^Department of Internal Medicine, College of Medicine, Hanyang University, Seoul, South Korea; ^5^Department of Music and Science for Clinical Practice, Hanyang University, Seoul, South Korea

**Keywords:** chronic liver disease (CLD), cognitive alteration, nonverbal auditory perception/music perception, melodic contour identification, haemodynamic response, functional near-infrared spectroscopy (fNIRS)

## Abstract

Early detection and evaluation of cognitive alteration in chronic liver disease is important for predicting the subsequent development of hepatic encephalopathy. While visuomotor tasks have been rigorously employed for cognitive evaluation in chronic liver disease, there is a paucity of auditory processing task. Here we focused on auditory perception and examined behavioral and haemodynamic responses to a melodic contour identification task (CIT) to compare cognitive abilities in patients with chronic liver disease (CLD, *N* = 30) and healthy controls (*N* = 25). Further, we used support vector machines to examine the optimal combination of channels of functional near-infrared spectroscopy that can classify cognitive alterations in CLD. Behavioral findings showed that CIT performance was significantly worse in the patient group and CIT significantly correlated with neurocognitive evaluation (i.e., number connection test, digit span test). The findings indicated that CIT can measure auditory cognitive capacity and its difference existing between patient group and healthy controls. Additionally, optimal subsets classified the 16-dimensional haemodynamic data with 78.35% classification accuracy, yielding markers of cognitive alterations in the prefrontal regions (CH6, CH7, CH10, CH13, CH14, and CH16). The results confirmed the potential use of behavioral as well as haemodynamic responses to music perception as an alternative or supplementary method for evaluating cognitive alterations in chronic liver disease.

## Introduction

Chronic liver disease (CLD) is a progressive destruction of liver functions over a period more than 6 months leading to fibrosis and cirrhosis (Sharma and Nagalli, 2020). Depending on the severity of impairment, patients with CLD show the limited mental capacity, changes in psychomotor functions and/or hepatic coma (Bernthal et al., 1987; Brodersen et al., 2014; Filipović et al., 2018). CLD-related neurophysiological and psychometric dysfunctions vary, ranging from psychomotor speed to executive functioning (Ortiz et al., 2005; Zhan and Stremmel, 2012) and get worsened as approach to overt hepatic encephalopathy (HE) (Butterworth, 2000; Sánchez-Carrión et al., 2008). Patients with HE present with overt clinical symptoms, such as disorientation, and consciousness disorders, which contributes to an increased risk of death in cirrhotic patients (Bustamante et al., 1999; Weissenborn et al., 2001a, 2005; Bajaj et al., 2007a; Prasad et al., 2007; Stewart et al., 2007). Early detection of cognitive alteration in chronic liver disease is, thus, critical to predict the subsequent development of HE (Krieger et al., 1996; Romero-Gómez et al., 2001; Chen et al., 2013).

The current cognitive evaluation in chronic liver disease do not cater for cognitive functions in diverse sensory modalities. For example, the West Haven scale is a subjective and semi-quantitative clinical scale that classifies mental state changes (Conn et al., 1977; Groeneweg et al., 1998; Hartmann et al., 2000; Ferenci et al., 2002; Amodio et al., 2004). In addition, there is a battery of psychometric tests that aims to detect neurocognitive impairments, such as neuropsychological and perceptual motor dysfunction (Weissenborn et al., 2005), named the psychometric hepatic encephalopathy score (PHES). The PHES consists of five subtests, including the A and B number connection tests (NCT-A, NCT-B), the line tracing test (LTT), the serial dotting test (SDT), and the digit symbol test (DST) (Weissenborn et al., 2001b; Ferenci et al., 2002; Bajaj et al., 2009), a primarily paper-and-pencil test. The stimuli in these neurocognitive tasks are limited to visual perception and visuomotor agility and rarely provide information about cognitive alteration in auditory modality. Clinicians and researchers have emphasized to a novel method that employs another type of stimuli and tasks to detect cognitive alterations, and that is also time- and cost-effective (Bajaj et al., 2007b, 2008; Romero-Gómez et al., 2007; Sharma et al., 2013; Kircheis et al., 2014; Gupta et al., 2015), which is a central aim of this study.

In this study, we focused on the potential of auditory perception as a new evaluation task (Mehndiratta et al., 1990; Saxena et al., 2001). For instance, Mehndiratta et al. (1990) employed different modality tasks to detect hepatic encephalopathy (HE), auditory task as measured by brain stem auditory evoked potentials were found to be the most sensitive to indicate HE compared to visual and somatosensory evoked potentials. Event-related potentials (ERPs) using an auditory oddball test have been shown to have significantly delayed P300 components in patients with minimal hepatic encephalopathy (MHE, the earliest form of HE characterized by neurocognitive impairment; Stinton and Jayakumar, 2013) and in patients with cirrhosis compared to healthy adults (Ciećko-Michalska et al., 2006; Teodoro et al., 2008). In a similar vein, Moon et al. showed cirrhotic patients had longer latencies for N100, P200, N200, and P300 than healthy adults in the auditory oddball test (Moon et al., 2014). In particular, the N200 latency, a negative peak related to mismatch detection and executive cognitive control function (Folstein and Van Petten, 2008), was significantly prolonged in cirrhotic patients than in healthy adults. The authors suggested that both P300 and N200 were delayed due to a slowness in intracerebral nerve conduction and that the two EEG components can be considered to be the first signs of cerebral deterioration in HE (Moon et al., 2014).

Rather than the auditory oddball test, in which target and non-target stimuli are presented consecutively, this study employed melodic contour identification task (CIT) that is designed to measure the selectivity of auditory attention, which is the core characteristics of auditory information processing. Our previous studies confirmed the validity of using melodic CIT to measure the various types of attention in moderate-to-severe traumatic brain injury patients. Jeong (2013) validated that the melodic contour stimuli could distinguish the different types and capacities of auditory attention existing in the various age groups and that it is a valid and reliable test for auditory cognition. They also that melodic CITs can measure attentional and cognitive dysfunctions existing clinical populations (Jeong and Lesiuk, 2011). More recently, the updated and computerized version of the test was scaled up, showing that the different CITs could distinguish different cognitive loads (Jeong et al., 2018).

We also examined HbO_2_ (oxygenated hemoglobin) in the frontal lobe, which is known as an indicator of cognitive alterations in patients with chronic liver disease (Mendonça et al., 2013). Keiding and Pavese (2013) revealed that the cerebral oedema yields dysfunction in haemodynamic responses and, thus, it is the main cause of cognitive alterations in HE. Macias-Rodriguez et al. (2016) supported the idea that the severity of HE contributes to haemodynamic alteration, mainly caused by damage to the vascular system and dysfunction of auto-regulatory vascular responses. The prefrontal areas modulate diverse cognitive systems, such as attention, working memory, decision-making, and problem solving (Amodio et al., 2004; Felipo et al., 2012; Jao et al., 2015) and to receive projections from almost all processing levels in the superior temporal gyrus (Poremba et al., 2004; Kusmierek and Rauschecker, 2009; Kikuchi et al., 2010). With regards to oxygen changes, to a lesser extent, prefrontal activity has been examined with patients with MHE. To our best knowledge, there existed a single study examining oxygen consumption changes during cognitive performance at the prefrontal cortex using fNIRS. Nakanishi et al. (2014) compared regional HbO_2_ in cirrhotic patients without MHE and those with MHE during a word fluency task. Their findings showed a significant difference between groups in HbO_2_ changes over time. That is, HbO_2_ in the MHE group were gradually increased throughout tasks, while the non-MHE group showed recurrent patterns of abrupt increase and decrease in concentrations. Also, the increase in HbO_2_ upon stimulation was significantly delayed in the MHE group than in non-MHE group (i.e., 5 s after stimulus presentation). These findings were suggestive of HbO_2_ obtained from the frontal area can reflect cognitive alterations along with progress of liver disease.

Thus, the main purpose of this study was to examine the potential of the melodic CIT as a test for cognitive alterations following chronic liver disease. We measured behavioral performance using three subtests of the CIT (focused, selective, and alternating listening, respectively) and examined the criterion validity of CIT, in correlation with standard neurocognitive tests. We also examined whether changes in HbO_2_ can be indicative of cognitive alterations in chronic liver diseases, and whether the regions of the prefrontal cortex, known to modulate attention and cognition, can be specified to characterize the alterations in chronic liver disease. For this analysis, we employed a support vector machine (SVM) combined with an fNIRS to classify cognitive alterations following liver diseases, a technique which has been increasingly used to classify clinical and healthy populations. Monden et al. (2015) previously measured HbO_2_ during a go/no-go task and performed classification using a SVM algorithm. The findings of that algorithm yielded a 90% accuracy, indicating the potential of SVM as a tool for classifying and, thus, diagnosing children with attention deficit hyperactivity disorder (ADHD). In addition, Ichikawa et al. (2014) employed an exhaustive search method combined with SVM that explored all possible combinations of the fNIRS channels to classify children with ADHD and autism spectrum disorder (ASD).

## Methods

### Participants

A total of 55 participants were included: 30 patients with chronic liver diseases (Male = 19, Female = 11), and 25 healthy controls (Male = 8, Female = 17) were matched by age and the level of education. Participants who had a <3 months of regular involvement in musical activities and/or professional training and who had a minimal ability to understand the spoken instruction were eligible to participate in the study. The diagnosis of liver disease was based on either a liver biopsy or the presence of portal hypertension and markers of hepatocyte synthetic dysfunction. We included patients who have been diagnosed as liver disease for more than 6 months. Participants with any of the following conditions were excluded: diabetes mellitus, systemic arterial hypertension, metabolic liver disease (hemochromatosis and Wilson's), personal history of stroke or cancer, use of neuropsychiatric drugs, neuropsychiatric disorders, current alcohol intake or smoking, rotating shift work, or acute inflammatory responses of infectious origin. Table 1 presents the demographics of the two groups.

**Table 1:** Participant demographics.

	**CL (*****N*** **=** **30)**		**HC (*****N*** **=** **25)**		***p*-value**
	**Mean**	***SD***	**Mean**	***SD***	
Age (years)	55.80	6.91	54.64 ±	5.94	0.205
Education (years)	10.80	3.67	13.48 ±	3.10	0.394

*CL, Patients with chronic liver disease; HC, Healthy control group*.

*N = 55 for this correlation analysis*.

### Musical Stimuli and Tasks

**Figure 1:**
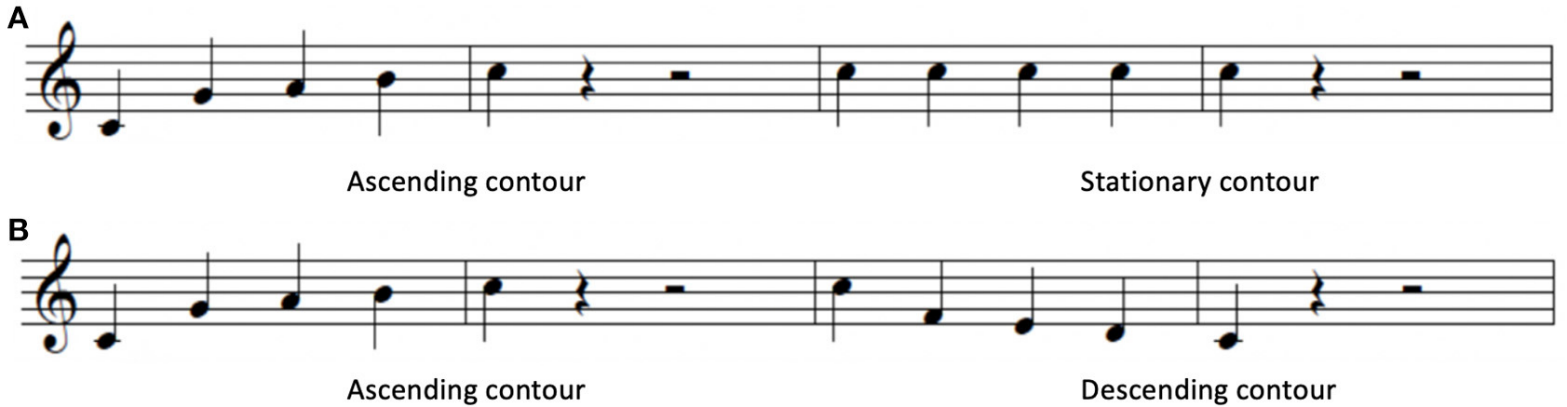
Sample melodic contours. **(A)** Ascending and stationary contours, **(B)** ascending and descending contours.

Three melodic contours (ascending, stationary, descending) were adopted from Jeong and Ryu (2016). Melodic contours are a series of tones moving in different directions (i.e., ascending, descending, and stationary). Two different types of contours were combined consecutively to yield six test items (i.e., ascending and descending, ascending and stationary, stationary and ascending, stationary and descending, descending and ascending, descending and stationary). The presentation time of each test item was 5,250 ms, including two contours (2,250 ms for each) and inter-contour interval (750 ms). Figure 1 shows examples of the pitch contours used in the study.

The six test items were modulated in five different keys (G# to C major) and were presented using three instrument timbres (i.e., piano, flute, string), yielding a total of 90 test items. We selected the instruments based on a previous study that classified various musical instruments according to their spectral features of timbre, such as the harmonic structure, inharmonicity, and harmonic energy skewness (Agostini et al., 2003). The melodic contours were generated by a digital audio workstation (Logic Pro X, Apple Inc., Cupertino, CA, USA) with amplitude normalization. The experimental test was developed as a computerized version, using Visual Studio (Microsoft, Washington, DC, USA).

Contour identification tasks (CITs). The computerized version of the CIT was designed to measure different types of auditory attention and the associated cognitive load changes (Table 2). The task stimuli and task structures were adopted from previous studies (Jeong, 2013; Jeong and Ryu, 2016; Jeong et al., 2018) and modified for the current purpose. In CIT1, two consecutive contour directions were presented as target contour with environmental sounds, including traffic, raining, twittering, ticktack, bustling, laughing, gabbling, applause, crying, and jeering sounds. They were randomly presented against target contours (i.e., selective contour identification against environmental noise). In CIT2, participants were presented with target melodic contours against target-like distractors (i.e., another melodic contour played by different instrument timbres) and were asked to identify a target contour presented in a predetermined instrument timbre. In CIT3, two melodic contours were presented, while participants were asked to shift their attention from one to another target contour and identify the direction of contours. For both CIT2 and CIT3, a visual cue (e.g., a picture of an instrument) was shown on the computer screen to inform about the instrument timbre of target contour. For all CITs, the direction of melodic contours and instrument timbres were randomly selected.

**Table 2:** Structure of the CITs.

	**Target**	**Distractor**	**Given task**	**Cognitive load**
CIT1	Melodic contour	Environmental sounds	Selective identification-Basic	Low 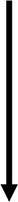 High
CIT2	Melodic contour	Target-like contours	Selective identification-Advanced	
CIT3	Melodic contour	Target-like contours	Alternating	

*CIT, contour identification task*.

**Figure 2:**
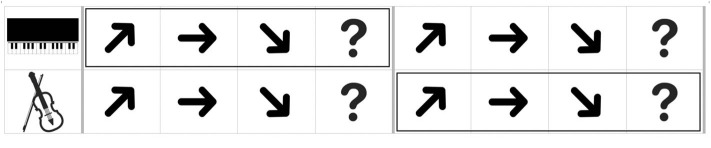
An example of an answer page given in a monitor with the musical stimuli (CIT3). The boxes were presented prior to presentation of each contour.

CIT1 had no visual cues, however, in CIT2, a picture of an instrument that plays target contours was presented prior to presenting the item to inform which contour the participants selectively listened to. In CIT3, outlined boxes were additionally used to guide at which contours the participants selectively listened to and shifted from one to another instrument (see Figure 2). For example, the first outlined box appeared in the upper or lower line with the first set of contours, and the second box appeared with the second set of contours.

### Haemodynamic Measurements

**Figure 3:**
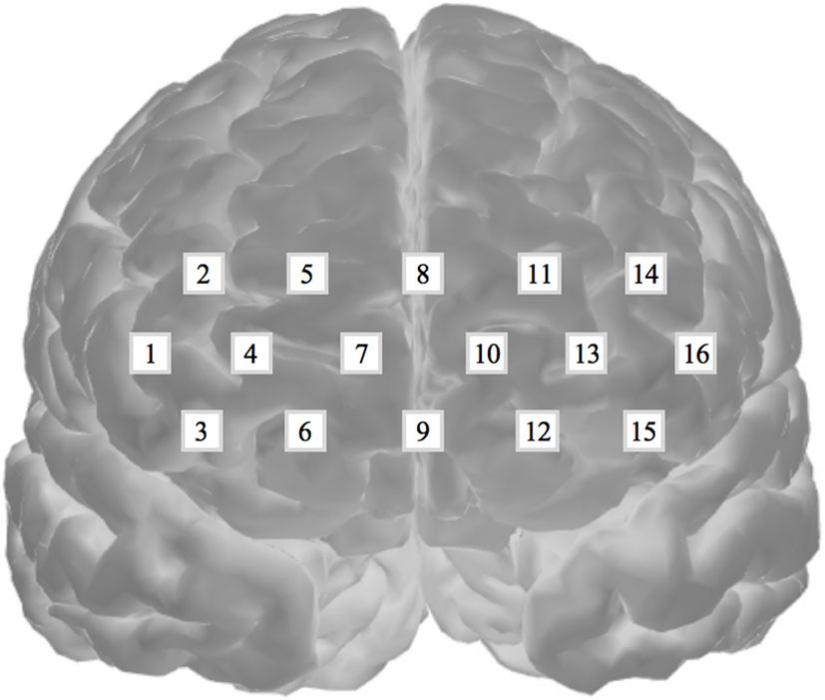
Spectratech OEG-16. The locations of the 16 fNIRS channels along the frontal cortex. The center of the measurement unit was placed on the frontopolar (Fp) region according to the international 10–20 system.

Oxygenated hemoglobin was measured to evaluate cognitive activation in participants and the loads imposed by the given tasks (Peck et al., 2013; Ogawa et al., 2014; Yasumura et al., 2014). For this evaluation, we employed an fNIRS and a non-invasive to monitor cortical tissue oxygenation (oxygenated hemoglobin, HbO_2_; deoxygenated hemoglobin, HHb) during cognitive, motor, and sensory stimulation. We used a 16-channel Spectratech OEG-16 (Shimadzu Co. Ltd., Kyoto, Japan) for the measurements (Figure 3). Task-related haemodynamic changes in HbO_2_ were recorded in 16 channels with a sampling rate of 0.65 s. In addition to the fNIRS data, we collected behavioral data, including task performance accuracy and reaction time.

### Experimental Procedure

This study was approved by the Institutional Review Board of Hanyang Medical Centre (HYUH-2013-08-017-002). Healthy adults voluntarily participated and were recruited *via* physical and online advertisements and patients with CLD were recruited *via* Hanyang Medical Center. All participants gave written informed consent in accordance with the Declaration of Helsinki. All other experimental methods were performed in accordance with the relevant guidelines and regulations. Prior to the experiment, two medical doctors with 17 and 20 years of experience, respectively, met with the participants to determine the participation eligibility and administered neurocognitive tests.

**Figure 4:**
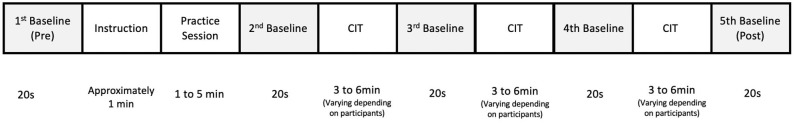
The flow of the experiment.

After taking a 30-min break, the participants wore a band-type fNIRS containing an array of 12 probes on their forehead. Participants had a short rest period and then the pre-stimulus baseline data were obtained for 20 s while they fixated their eyes on the center of the monitor. A 20-s baseline was also obtained during inter-task rest periods and post-task period. Once the baseline data were obtained, the participants were presented with instruction and examples of melodic contours. Each of the three CITs started with brief instruction in terms of the task characteristics given in each CIT and how to respond to test items. Participants were also instructed to identify the directions of the target contours by clicking the arrow corresponding to the contour direction as accurate and immediate as possible. The contours were delivered *via* a headphone with controlled volume, while the visual cues specifying the target musical instrument were presented to the participants on a monitor. The participants underwent a practice session to become familiar with the direction identification task. When their accuracy was over 80%, the main experimental session was administered. A total of 18 test items were presented in each CIT (a blocked design) and the order of CIT was randomized across participants (Figure 4). The CITs took about 20 min to complete. The experiment was performed in a sound-proof room, in which light and temperature were controlled.

### Signal Pre-processing

The fNIRS raw data were collected throughout the experiment and were converted into concentration changes of hemoglobin using the modified Beer–Lambert law (Baker et al., 2014). Subsequently, a zero-phase low- and high-pass filter with a cut off frequency from 0.01 to 0.09 Hz was applied to remove any noise from heartbeat pulsations and longitudinal signal drifts (Morren et al., 2004; Akgül et al., 2005; Bauernfeind et al., 2011). In this study, we employed an HbO_2_ index that was based on previous studies, which reported that HbO_2_ shows better classification performance than other measures (Li et al., 2015), especially for conditions with a high-dimensional feature and low sample sizes (Mourao-Miranda et al., 2005; Yoon et al., 2007). The obtained HbO_2_ values were standardized by subtracting the mean of 20-s pre-stimulus baselines in order to compare directly across participants and channels (Herff et al., 2013). Subsequently, the mean HbO_2_ during each CIT (CIT1, CIT2, CIT3) was calculated for each of the 16 channels. Since we found that the fNIRS response peak was delayed by a few seconds compared to the stimulus onset (Cui et al., 2010; Ichikawa et al., 2014), we eliminated the HbO_2_ from the first 3–10 s for the statistical analysis. Finally, we obtained 165 data points, including 90 from CL (including 30 from the MHE subgroup) and 75 from HC. We used the data as inputs and the diagnosis (HC, CL, MHE) of the groups as outputs.

### Classification Using Support Vector Machines (SVMs)

In this study, we employed a linear SVM model with a repeated k-fold **cross** validation to solve classification problems between HC and patients with liver disease. K-fold cross validation method is one of the split sample methods that randomly divide data into *k* subsets, then one of the *k* subsets are used as the test set and the other k-1 subsets are used as training set. This process is iterated *k* times so that each subset is used as testing set then results are averaged (Kim, 2009; Mishra and Sahu, 2011). We adopted 5-fold cross validation with repeated 20 times to accurately estimate the generalized performance of the classification (Refaeilzadeh et al., 2009). Then, we evaluated classification performance using the MCC and the bACC, as described by previous studies (Sakiyama et al., 2008; Jiao and Du, 2016). The studies recommended the use of different evaluation methods to confirm high classification performance in practical application (Jiao and Du, 2016).

**Figure 5:**
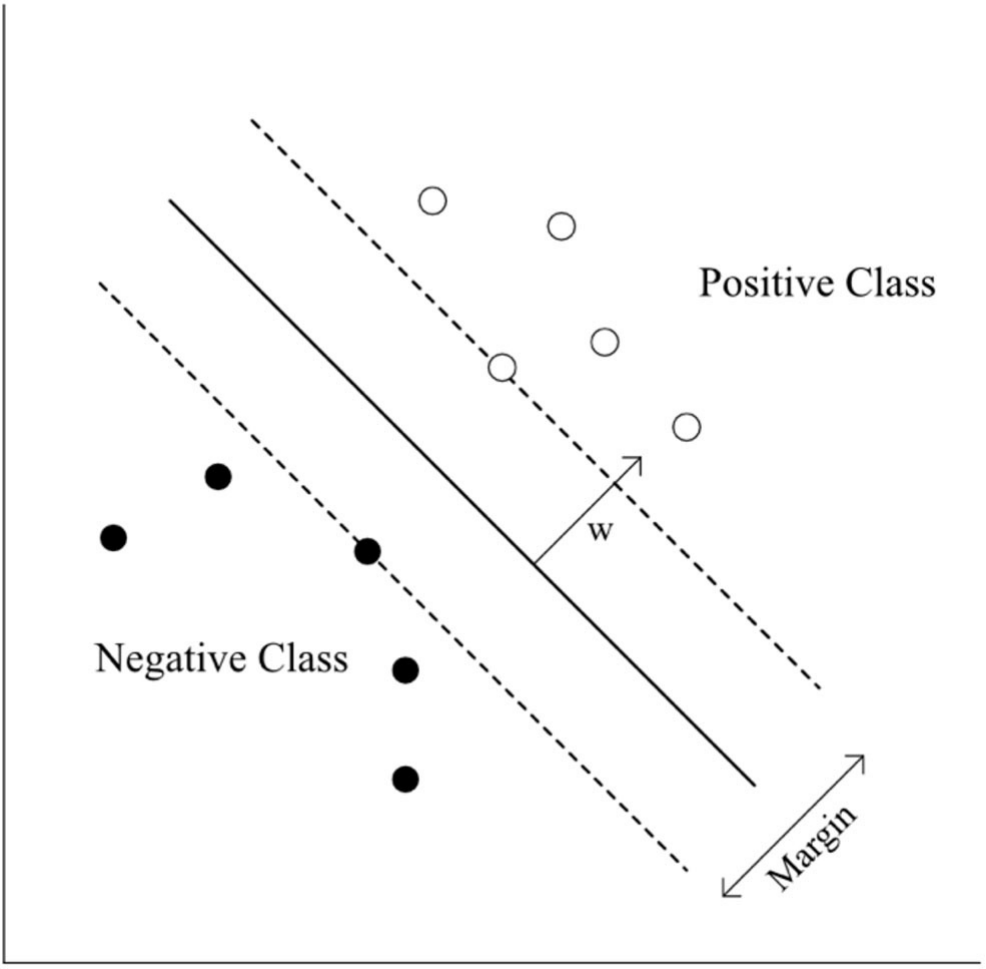
SVM classification.

The standardized mean of HbO_2_ was trained in three different ways, including (1) eight channels from the right hemisphere (CH1–CH8), (2) eight channels from the left hemisphere (CH9–CH16), and (3) 16 channels from both hemispheres (CH1–CH16). The standardized HbO_2_ data from the eight channels from both hemispheres and the 16 channels have an 8-dimensional (2^8^- 1 = 255 subsets) or 16-dimensional (2^16^- 1 = 65,565 subsets) feature vector, respectively. The obtained HbO_2_ data were trained and classified to diagnose groups. SVMs have been applied previously for classification problems in various domains, and have yielded high generalization capability (Bennett and Campbell, 2000; Lotte et al., 2007). An SVM learns the relationship between the input and output, (i.e. classes) from the given set of data. In the feature vector space (Figure 5), the SVM algorithm creates a hyperplane that separates the input data into two classes with a maximum margin.

The following equation describes the hyperplane of SVM.

(1) $$\begin{array}{l} {\text{w}}^{\text{T}}\text{x}+\text{b}=0, \end{array}$$

Where **w** is the normal vector to the hyperplane, the two classes are represented as follows:

(2) $$\begin{array}{l} {\text{w}}^{\text{T}}{\text{x}}_{\text{i}}+\text{b}\geq 1\text{ for }{\text{y}}_{\text{i}}=1, \end{array}$$

(3) $$\begin{array}{l} {\text{w}}^{\text{T}}{\text{x}}_{\text{i}}+\text{b}\leq -1\text{ for }{\text{y}}_{\text{i}}=-1, \end{array}$$

$$\begin{array}{l} {\text{x}}_{\text{i}}=\left({\text{x}}_{\text{i}1},{\text{x}}_{\text{i}2},\ldots ,{\text{x}}_{\text{in}}\right),{\text{y}}_{\text{i}}\subset \left\{1,\;-1\right\}, \end{array}$$

where **x**_i_ is the i^th^ example of the training data and y_i_ is its label.

Once we trained the SVM model, the weight vector **w** is known. To test the model, we put a test sample into the left side of the **Equation (1)**. If the value was more than 0, the sample was classified as positive. If the value was <0, the sample was classified as negative.

### Evaluation

To evaluate the trained model, we first computed the confusion matrix shown in Table 3. A confusion matrix describes the classification result of the test samples by counting the number of true positives, true negatives, false positives, and false negatives. If both actual and predicted classes of a sample are positives, the sample is a true positive; similarly, the sample is a true negative when both classes are negatives. If the actual class of a sample is positive but the predicted class of the sample is negative, then the sample is a false negative. If the actual class of a sample is negative but the predicted class is positive, the sample is a false positive.

**Table 3:** Confusion matrix.

		**Predicted Class**	
		**Positive**	**Negative**
Actual Class	Positive	True Positive (TP)	False Negative (FN)
	Negative	False Positive (FP)	True Negative (TN)

From the confusion matrix, the basic performance measures were calculated according to the following equations:

(1) $$\begin{array}{l} \text{Sensitivity}:\text{TP}/\left(\text{TP}+\text{FN}\right) \end{array}$$

(2) $$\begin{array}{l} \text{Specificity}:\text{TN}/\left(\text{TN}+\text{FP}\right) \end{array}$$

(3) $$\begin{array}{l} \text{Accuracy}:\left(\text{TP}+\text{TN}\right)/\left(\text{TP}+\text{FP}+\text{FN}+\text{TN}\right) \end{array}$$

Sensitivity is the probability to correctly predict the samples that are actually classed as positive. It is an indicator that assess the ability of a model to identify positive samples. Specificity also assess a model's ability to correctly to correctly predict the samples whose class is actually negative. Accuracy is the ability of a model to correctly identify classes for all samples. However, accuracy could lead to evaluation errors when the data is imbalanced. If one class is a majority class, the real performance of the other class cannot be reflected accurately. Moreover, sensitivity and specificity are not proper measures to study the balanced performance. For a balanced evaluation, the following measure was used.

(4) $$\text{MCC}=\frac{\text{TP}\cdot \text{TN }-\text{ FP}\cdot \text{FN }}{\surd (\text{TP}+\text{FP})(\text{TP}+\text{FN})(\text{TN}+\text{FP})(\text{TN}+\text{FN})}$$

(5) $$\begin{array}{l} \text{bACC}=\left(\text{Sensitivity}+\text{Specificity}\right)/2 \end{array}$$

We assessed a modified classification performance measure using a MCC, which is used widely in biomedical research (Van't Veer et al., 2002; Boughorbel et al., 2017). MCC considers all of the confusion matrix categories (true positives, true negatives, false positives, false negatives), making it suitable for providing a more balanced value when the sample numbers between groups were imbalanced (Brodersen et al., 2010; Powers, 2011). Because of a difference in the number of samples in our patients (CL = 30, and HC = 25), we adopted the MCC to classify performance measures. MCC values range between −1 and +1. If an MCC coefficient is +1, it means the classifier can perfectly predict the class of the data. An MCC coefficient = 0 means that it is not different from a random prediction, and a −1 coefficient means it totally mispredicts the class. Also, we considered the bACC, which is the average of sensitivity and specificity since it is also one of the ways to solve an imbalanced dataset (Brodersen et al., 2010; Powers, 2011), for our accuracy measurements. All statistical procedures were performed using R.

## Results

We analyzed behavioral responses to CITs and performed classifications between patients with chronic liver disease (CL) and healthy controls (HC) using HbO_2_. HbO_2_ was obtained from 16 fNIRS channels located in the prefrontal cortex. We employed SVM with the exhaustive search method and grouped three data sets, including (1) 8-dimension dataset obtained from the right hemisphere (CH1–CH8), (2) 8-dimension dataset obtained from the left hemisphere (CH9–CH16), and (3) 16-dimension dataset obtained from the bilateral hemispheres (CH1–CH16). For each of these three datasets, we trained the SVM and evaluated its classification accuracy using Matthew correlation coefficient (MCC) and the balanced accuracy (bACC).

### Behavioral Responses

Table 4 shows the mean accuracy and reaction time for the CL and HC groups on the CIT. The mean accuracy in the HC group was the highest for CIT1(0.75), and was followed by the CIT2 score (0.48). Accuracy in the HC was lowest (0.46) when an attention shift was required between two concurrent melodic contours during CIT3. A similar trend was found across the CITs for the CL group (decreasing from 0.62 to 0.32). However, accuracy declined considerably between both the CIT1 and CIT2 and between the CIT2 and CIT3, while the HC group showed an obvious decrease only between CIT1 and CIT2. Reaction time showed a similar trend between HC and CL groups, and was the shortest in the CIT1 and the longest in the CIT3.

**Table 4:** Statistical difference of CIT performance between groups.

	**Accuracy**				**Reaction time**			
	**CL (*****N*** **=** **30)**		**HC (*****N*** **=** **25)**		**CL (*****N*** **=** **30)**		**HC (*****N*** **=** **25)**	
	***M***	***SD***	***M***	***SD***	***M***	***SD***	***M***	***SD***
CIT1	0.62^a^	0.30	0.75^d^	0.28	8576.44^f1^	816.34	5799.34	894.26^h1^
CIT2	0.45^b^	0.23	0.48^e1^	0.25	9595.72^f2^	1154.55	6695.61	1264.74^h2^
CIT3	0.32^c^	0.21	0.46^e2^	0.26	11139.09^g^	1490.87	6926.38	1633.17^h3^

*CL, Patients with chronic liver disease; HC, Healthy control group; M, Mean; SD, Standard Deviation*.

*Different superscript letters in the same column indicate statistical significance*.

*p < 0.001 for the pairs of a and b, b and c, c and a, d and e^1^, d and e^2^*.

*p < 0.05 for the pairs of f1 and g, f^2^ and g. The pairs of e^1^ and e^2^, f^1^ and f^2^, h^1^, h^2^, and h^3^ did not show any statistical significance (p > 0.05)*.

A two-way mixed ANOVA [i.e., Group (CL, HC) × Task (CIT1, CIT2, CIT3)] was performed to validate accuracy and reaction time. For accuracy, there was a significant main effect of the Task [*F*_(2, 110)_ = 65.566, *p* < 0.001], indicating that CIT performance for both groups worsened significantly as the CITs became more difficult. The pairwise *post hoc* comparison using the Bonferroni correction revealed that CIT1 was significantly better than CIT2 (*p* < 0.001), and that CIT2 was significantly better than CIT3 (*p* < 0.01). We also found a significant interaction between the Group and Task [*F*_(2, 110)_ = 3.114, *p* < 0.05]. Interestingly, the pairwise *post-hoc* comparison revealed that the CL group performed significantly better in the CIT2 than in the CIT3 (*p* < 0.001), while the HC group showed similar performances in the CIT2 and CIT3 (*p* > 0.05). There was a significant main effect of the Task [*F*_(2, 110)_ = 6.126, *p* < 0.01] on the response time. The pairwise *post hoc* comparison using the Bonferroni correction revealed that only the CL group performed significantly worse in the CIT3 than CIT1 and CIT2 (*p* < 0.05, respectively). Table 4 present the statistical difference of behavioral performance between groups. Our behavioral findings collectively indicated that patients with chronic liver disease showed significantly worse auditory perception and cognition, specifically between CIT2 and CIT3. Additionally, we performed a correlation analysis to examine the criterion validity of the CITs. Table 5 presents the correlations between the CITs and neurocognitive evaluations.

**Table 5:** Correlation between CIT and neurocognitive evaluation tests.

		**CL**				**HC**			
		**NCT-A**	**NCT-B**	**DST-Forward**	**DST-Backward**	**NCT-A**	**NCT-B**	**DST-Forward**	**DST-Backward**
Accuracy	CIT1	−0.673^**^	−0.546^**^	0.32	0.325	−0.3	−0.470^*^	0.560^**^	0.558^**^
	CIT2	−0.565^**^	−0.520^**^	0.38	0.33	−0.438^*^	−0.429^*^	0.466^**^	0.398^*^
	CIT3	−0.593^**^	−0.459^*^	0.415^*^	0.367	−0.418^*^	−0.473^**^	0.521^**^	0.414^*^
	Total	−0.691^**^	−0.573^**^	0.416^*^	0.383	−0.409^*^	−0.499^**^	0.566^**^	0.509^**^
Reaction time	CIT1	0.753^**^	0.567^**^	−0.248	−0.332	0.752^**^	0.759^**^	−0.431^*^	−0.304
	CIT2	0.670^**^	0.576^**^	−0.289	−0.231	0.626^**^	0.649^**^	−0.380^*^	−0.24
	CIT3	0.692^**^	0.566^**^	−0.386	−0.182	0.721^**^	0.714^**^	−0.467^**^	−0.319
	Total	0.752^**^	0.602^**^	−0.311	−0.279	0.723^**^	0.732^**^	−0.435^*^	−0.295

*NCT, Number Connection Test (Time to completion, sec); DST, Digit Span Test (Number of correct answers)*.

*N = 55 for this correlation analysis*.

** *p < 0.01*,

* *p < 0.05*.

### Classification of Hemodynamic Responses

Further, we classified the differences in haemodynamic responses to CIT1 and CIT2 between HC and CL in combination with an SVM and an exhausted feature selection method. We decided to exclude CIT3 since our behavioral findings clearly showed that CIT3 was too difficult for CL group and, thus, haemodynamic response to CIT3 might not indicate appropriate cognitive load in this group. With the analysis, we found a total of 15 subsets that classified the data more accurately into two groups among the 255 subtests (five each for the right, left, and bilateral hemispheres, respectively). Table 6 presents the classification performance for each of the 15 subsets. The classification performance was higher for the right hemisphere (MCC = 0.451) than the left hemisphere (MCC = 0.317). The compute score of sensitivity and specificity was also higher for the right hemisphere (bACC = 69.75%) than in the left (bACC = 64.50%) hemisphere. The classification performance was best with CH6, CH7, CH10, CH13, CH14, and CH16 (MCC = 0.577, bACC = 78.35%), indicating that the inclusion of HbO_2_ obtained from the bilateral hemisphere yielded better classification performance than values obtained from the unilateral hemispheres. After an additional *t*-test, we found that classification performance of the bilateral subset was significantly higher than that of the right and left hemisphere subsets (*p* < 0.001, respectively).

**Table 6:** Classification accuracy between HC and CL data sets.

**Hemisphere**	**Best subset**	**MCC**	**bACC**
Right	2,7	0.451	69.75%
	3,4,7	0.435	71.05%
	1,2,4,7,8	0.366	69.40%
	3,4,6,7	0.364	68.75%
	2,4,6,7	0.363	62.25%
Left	9,13,16	0.317	64.50%
	9,13	0.309	63.65%
	9,13,15	0.296	62.65%
	9,12,13,14,15	0.274	60.80%
	9,10,13	0.245	61.50%
Bilateral	6,7,10,13,14,16	0.577	78.35%
	6,7,10,12,13,14,16	0.575	78.00%
	1,2,6,7,12,13,16	0.571	77.45%
	4,6,7,9,13,14,16	0.560	77.45%
	4,6,7,8,13,14,16	0.548	76.70%

*N = 255 subtests for the right and left hemispheres, N = 65565 subtests for the bilateral hemisphere. Best subsets indicated a group of the channels that yielded optimum classification accuracy. MCC, Matthew Correlation Coefficient; bACC, balanced Accuracy*.

**Figure 6:**
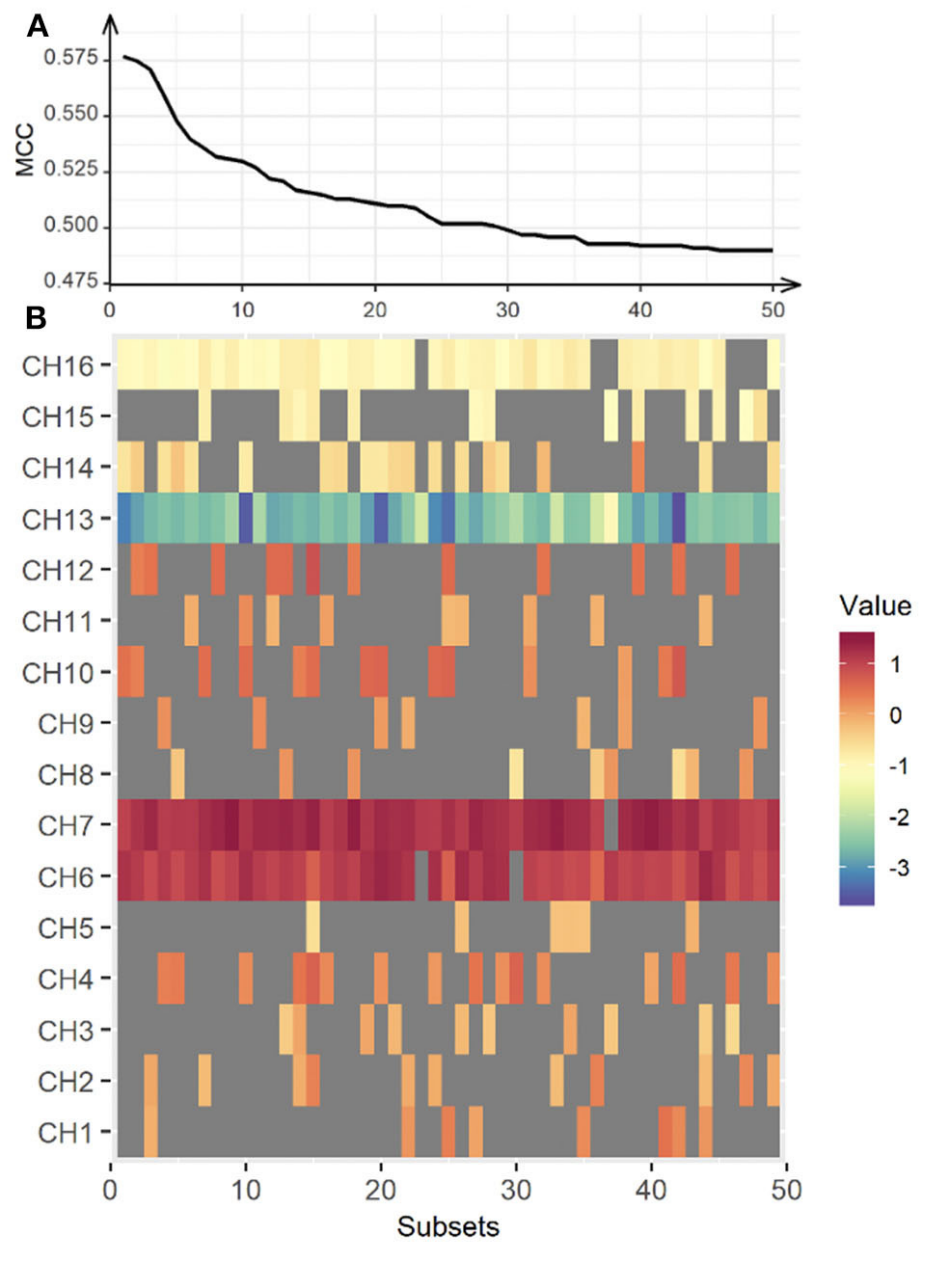
Classification performance for the bilateral hemisphere between CL and HC. The upper panel **(A)** represents classification performance for the best 50 out of 65,565 subsets. The lower panel **(B)** shows the weight vector of each channel matched in the subsets. MCC, Matthew Correlation Coefficient. Red indicates higher HbO_2_ in the CL, while blue indicates higher HbO_2_ in the HC.

Further, we created a matrix plot using 50 subsets and constructed features that correspond to the classification performance of each subtest. Figure 6 shows that CH6, CH7, CH10, CH13, CH14, and CH16 are effective features. This indicated that HbO_2_ in CH6, CH7, and CH10 were higher in the CL than in the HC, and that the HbO_2_ in CH13, CH14, and CH16 were lower in the CL than in the HC. Further, our findings suggest that the frontal areas of each hemisphere were intercommunicating, so it was necessary to include subsets from both hemispheres to yield better classification performance.

## Discussion

**Figure 7:**
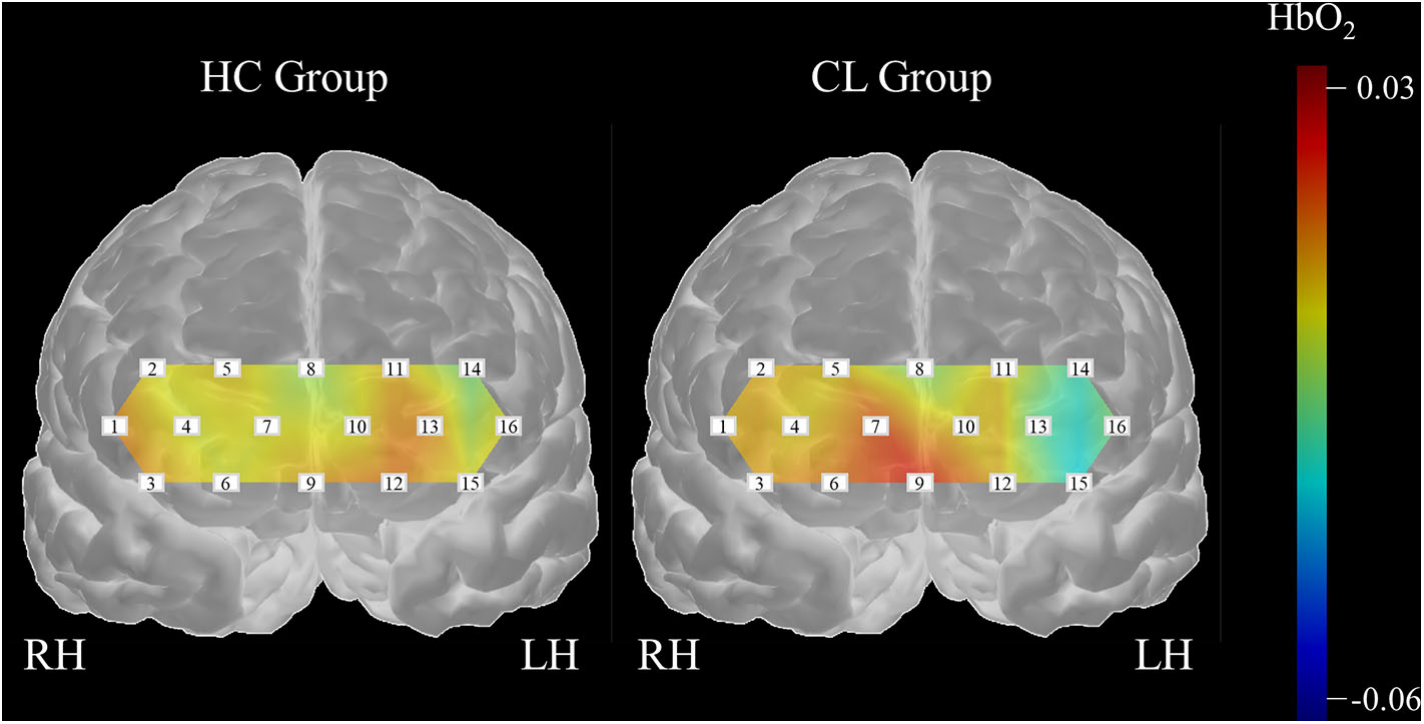
Differences of haemodynamic responses between the HC and CL groups.

In this study, we examined the potential of melodic CITs to evaluate cognitive alterations in chronic liver disease. Our behavioral findings indicated that CITs can differentiate changes in auditory perception and cognition in patients with chronic liver disease from healthy controls. Correlation analysis using standard neurocognitive evaluations revealed that CITs had good criterion validity and potential for measuring cognitive alterations that occur in chronic liver disease. Then, we applied SVMs with 5-fold cross validation to the haemodynamic responses obtained during the CIT performance to classify cognitive alterations in patients with chronic liver disease. We exhaustively searched all subsets of the measurement channels and evaluated each classification performance by repeating the 5-fold cross validation method 20 times. Our findings yielded an optimal subset for the classification of the haemodynamic data with 78.35% accuracy. Our results indicated that the subsets obtained bilaterally can better classify the differences that exist between HC and patients with CL. Also, we found channel features that could specify between groups. Three channels (CH13, CH14, CH16) in the left dorsolateral prefrontal cortex (DLPFC), one channel (CH6) in the right orbitofrontal cortex (OFC), and two channels (CH7, CH10) in the right frontopolar area (FP) were important for the classification of CL from HC. Figure 7 summarizes the channel specific findings and clearly shows haemodynamic difference existing between groups.

From the behavioral findings, we found poorer overall CIT performance in the CL than in the HC group, and the difference was more obvious between CIT2 and CIT3. The findings indicated that overall auditory cognition deteriorated in the CL group. Given that CIT1 and CIT2 involve auditory selective attention and that CIT3 involves auditory alternating attention, the cognitive threshold of patients with CLD might be auditory alternating attention (or cognitive flexibility). The current findings resembled previous findings where selective attention (Felipo et al., 2012) and cognitive flexibility (Yang et al., 2018) deteriorated in patients with liver-related diseases. The tasks employed in the previous studies are visual-oriented, so our findings revealed that the deterioration of selective attention and of cognitive flexibility exists similarly in an auditory modality.

Additional correlation analyses indicated that CIT can measure modality-general cognitive abilities and evaluate auditory cognitive deficits in chronic liver disease. In the CL and HC groups, the NCT-A and NCT-B highly correlated with the accuracy and reaction time of CITs. Correlations with DST were specific to groups. DST-Forward significantly correlated with accuracy and reaction time in the HC group, but this was not significant for the CL group (except for the accuracy of CIT3 and CIT total). DST-Backward significantly correlated with accuracy only in the HC group—not for the CL group. DST measures auditory working memory (DST-Forward) and auditory executive function (DST-Backward; Soltani et al., 2018). The current findings might indicate that the HC group utilized auditory working memory and executive function appropriately while performing CITs. The CL group, on the other hand, utilized these functions limitedly during CIT performance. The findings from correlation analyses further suggest that dysfunctions in higher auditory cognition (i.e., processing sequential auditory information) might be one of the characteristics of auditory cognitive deficits in the CL group.

From the classification of haemodynamic responses, we found that the channel features in this study were intriguing. Firstly, HbO_2_ values obtained from the left DLPFC (CH13, CH14, CH16) in the CL group were lower as compared to the HC group, indicating that patient group consumed less cognitive resources during the CIT performances than healthy adult group. DLPFC regions modulate higher order cognitive systems, such as working memory, decision-making, and problem solving (Amodio et al., 2004; Felipo et al., 2012; Jao et al., 2015). Also, this region is progressively impaired as liver disease advances (Chen et al., 2014), which can be a result of decreased blood flow and reduced glucose uptake activity in the frontal region (Lockwood et al., 1991, 2002). Ni et al. reported that patients with liver disease showed decreased functional coherence in the bilateral prefrontal cortex as the disease progressed (Felipo et al., 2012; Ni et al., 2012; Zhang et al., 2012). Taken together, deactivation in the left DLPFC observed in patients with chronic liver disease seemed to bring poor CIT performance, which can be indicative of cognitive alterations and inattention. Current inactivity of HbO_2_ in the DLPFC, thus, can be a useful feature to classify patients with chronic liver disease from healthy adults.

In addition, HbO_2_ values obtained from the right FP (CH7, CH10) were greater in the CL group as compared to HC group. The right frontopolar area, which corresponds to BA10, is involved in a variety of cognitive performances, ranging from simple to highly complex tasks (Burgess et al., 2007; Turner et al., 2008). This area receives projections from almost all processing levels in the superior temporal gyrus (Poremba et al., 2004; Kusmierek and Rauschecker, 2009; Kikuchi et al., 2010), and is known for its engagement in abstract representations of auditory information in organized thought (Medalla and Barbas, 2014). Further, lower activations in DLPFC and greater activation in FP in CL than HC group together indicated that healthy adults seemed to assign their cognitive resources on a higher-order cognitive function while patients with CL placed them in a more general attention and auditory cognition. The current findings possibly suggested a compensatory mechanism that reflects the recruitment and reallocation of cognitive resources due to the liver disease (Qi et al., 2012). Lastly, HbO_2_ values obtained from the CH6 were greater in the CL than HC group. CH6 receives signals from the FP (BA10), as well as the OFC (BA11). Note that BA11 is known for its role in emotional behaviors, especially in evaluating the emotional valence of external stimuli (Rolls, 2004; Powell et al., 2017). Huang et al. (2016) for example reported that the level of BA11 activation is indicative of an individuals' aesthetic experience. Greater activation CH6 in the CL than in the HC were, thus, possibly due to our participants' aversion or appetite for the auditory stimuli given in CITs rather than features of specific alterations that could be used to diagnose cirrhotic chronic liver disease.

## Limitation

The results of this study provide new information regarding the use multiple prefrontal area channels in the diagnosis of cognitive alterations in chronic liver disease. Our study preliminarily utilized auditory/music processing as an evaluation task in chronic liver disease, but it includes a small sample size (*N* = 55) and a skewed female-to-male ratio. Some previous studies have reported gender difference issues in patients with chronic liver disease (Tsai et al., 2015; Barreira et al., 2019). This issue remains controversial, and findings differ depending on the subtypes of cognitive functions and the types of tasks. In this study, the comparison of cognitive functions between males and females was limited by the insufficient sample size. In future upscaled studies, we will directly address this issue by controlling the sample size and gender ratio to investigate the possible influence of gender difference on cognitive functions in chronic liver disease. It is alsonecessary to confirm that auditory attention is affected in chronic liver disease and that it has potential as a biomarker for MHE detection.

Also, there is little doubt that the important aspects of attention and cognition are associated with other brain regions. As the full associations between attention and cognition were not comprehensively covered due to the physical limitations of our fNIRS device, the caveats of our study should be considered when inferring its relationship with overall activations in the cortex. Emergent advances in fNIRS technology that provide more channels should make it possible to cover more regions of the brain in future studies to examine compensatory mechanisms for cognitive alterations in chronic liver disease.

## Conclusions

In this study, we applied an SVM model with an exhaustive method, as suggested by Ichikawa et al. (2014), to classify the haemodynamic responses during auditory perception. This method was performed successfully and yielded chronic liver disease-specific channel features. Given that the majority of assessment stimuli are visual, these findings implicated the importance of auditory processing in evaluating cognitive alterations in chronic liver disease (Mehndiratta et al., 1990; Sawhney et al., 1997; Saxena et al., 2001; Moon et al., 2012). Second, by virtue of recent brain imaging technology, such as the fNIRS, changes in oxygenated hemoglobin in the prefrontal areas were examined in a time and cost-effective manner. There were several channels that differentiated group-specific cognitive alterations that were reflected in the auditory/music perception. The left DLPFC and frontopolar areas played a task-specific role, while the right DLPFC played a modality- and stimulus-specific role in classification. Lastly, SVMs combined with an exhaustive search method was effective in classifying multivariate haemodynamic data since it allowed us to extrapolate an optimized combination from all possible combinations. Auditory/music perception tasks identified cognitive alterations in chronic liver diseases, which is frequently observed but not yet clearly explained; thus, this method combined with CITs could potentially serve as a supplementary evaluation of cognitive functions in the early detection of HE.

## Data Availability Statement

The raw data supporting the conclusions of this article will be made available by the authors, without undue reservation, to any qualified researcher.

## Ethics Statement

The studies involving human participants were reviewed and approved by Institutional Review Board of Hanyang Medical Centre (HYUH-2013-08-017-002). The patients/participants provided their written informed consent to participate in this study.

## Author Contributions

GJ and EJ conceived and designed the experiments. DJ recruited participants and arranged individual experiments. EJ performed the experiments and edited the manuscript. GJ, Y-MK, DJ, and EJ analyzed, interpreted the data, and wrote the manuscript. GJ and EJ prepared tables and figures. All authors reviewed the manuscript.

## Conflict of Interest

GJ was employed by the company Daehong Communications Inc. The remaining authors declare that the research was conducted in the absence of any commercial or financial relationships that could be construed as a potential conflict of interest.
